# Aging Process of Biocomposites with the PLA Matrix Modified with Different Types of Cellulose

**DOI:** 10.3390/ma17010022

**Published:** 2023-12-20

**Authors:** Piotr Szatkowski, Jacek Gralewski, Katarzyna Suchorowiec, Karolina Kosowska, Bartosz Mielan, Michał Kisilewicz

**Affiliations:** 1 Department of Biomaterials and Composites, Faculty of Materials Science and Ceramics, AGH University of Krakow, Al. Mickiewicza 30, 30-059 Krakow, Poland; suchorowiec@agh.edu.pl; 2 Institute of Marketing and Sustainable Development, Lodz University of Technology, 93-590 Lodz, Poland; jacek.gralewski@p.lodz.pl; 3 Solaris National Synchrotron Radiation Centre, Jagiellonian University, Czerwone Maki 98, 30-392 Krakow, Poland; karolina.kosowska@uj.edu.pl; 4 Pre-Clinical Research Centre, Wroclaw Medical University, Bujwida 44, 50-345 Wroclaw, Poland; b.mielan@umw.edu.pl; 5 Technology Transfer Center, University of Applied Sciences in Tarnow, Ul. Mickiewicza 8, 33-100 Tarnow, Poland; m_kisilewicz@atar.edu.pl

**Keywords:** polylactide, wood, cellulose, flax, biocomposites, degradation, biodegradation, aging process

## Abstract

In the modern world, many products are disposable or have a very short lifespan, while at the same time, those products are made from materials that will remain in the environment in the form of waste for hundreds or even thousands of years. It is a serious problem; non-biodegradable polymer wastes are part of environmental pollution and generate microplastics, which accumulate in the organisms of living beings. One of the proposed solutions is biodegradable polymers and their composites. In our work, three types of polylactide-based composites with plant-derived fillers: microcellulose powder, short flax fibers, and wood flour at 2 wt.% were prepared. Poly(lactic acid) (PLA)-based biocomposite properties were characterized in terms of mechanical and surface properties together with microscopic analysis and Fourier-transform infrared spectroscopy (FTIR), before and after a UV (ultraviolet)-light-aging process to determine the effects of each cellulose-based additive on the UV-induced degradation process. This research shows that the addition of a cellulose additive can improve the properties of the material in terms of the UV-aging process, but the form of the chosen cellulose form plays a crucial role in this case. The testing of physicochemical properties demonstrated that not only can mechanical properties be improved, but also the time of degradation under UV light exposure can be controlled by the proper selection of the reinforcing phase and the parameters of the extrusion and injection molding process. The obtained results turned out to be very interesting, not only in terms of the cost reduction of the biocomposites themselves, as mainly the waste from the wood industry was used as a low-cost filler, but also that the additive delays the aging process occurring during UV light exposure. Even a small, 2 wt.% addition of some of the tested forms of cellulose delayed surface degradation, which is one of the most important factors affecting the biodegradation process.

## 1. Introduction

Limited resources of petroleum inspire the search for new raw materials to obtain polymers, especially from renewable sources. This is because plastics are mainly produced from fossil fuels (non-renewable sources like gas, coal, and oil) in contrast to bioplastics, e.g., PLA, which is produced from renewable plant-based resources [1,2]. Development in the bioplastic and biocomposite field impacts the reduction in carbon dioxide emissions and the improvement of polymer sustainability [3]. Governments require higher responsibility in managing resources and limiting the usage of conventional, e.g., PE (polyethylene) or PP (polypropylene), polymers for packaging applications [4]. For those reasons, there is a growing interest in green composites that contain at least one ingredient from renewable resources [5]. One of them is thermoplastic poly(lactic acid) (PLA). What makes it an attractive material as a composite matrix is that it does not increase CO_2_ emissions. Biocomposites made from plant sources initially have a net negative CO_2_ emission rate. During their lifetime, plants use CO_2_ as a substrate for the photosynthesis process; therefore, composites made from plant sources can exhibit a net negative CO_2_ emission rate because they absorb CO_2_ and through metabolic processes, it builds up in their tissues [6]. Nevertheless, the processing process into material, the conversion of plants into lactic acid and then into PLA, is an energy-intensive process that releases large amounts of CO_2_ into the atmosphere [7]. Additionally, sowing plants for the production of biodegradable plastics involves the use of pesticides, the use of agricultural land at the expense of edible plants, and water consumption, which has negative consequences for the environment and people [8]. To determine the environmental impact, every time we must consider raw material production, manufacturing processes, product use, end of life management, etc. PLA does and will have a leading role in the sustainable plastic economy, but it is not a panacea.

The life cycle of biodegradable polymers is much more favorable in terms of energy consumption for its production compared to PE (polyethylene); additionally, GHG (greenhouse gasses) emission is two-fold lower and the energy consumption in its production process is three-fold lower [9]. Additionally, PLA may be reused because it undergoes hydrolysis and then resynthesis during the degradation and further absorption by the plants [3]. Its properties strongly depend on many parameters like the chirality of monomers, molecular weight, crystallinity, etc., but it may resist stress in the range of 45–70 MPa during tensile strength tests, and be applied in, e.g., 3D printing or packaging [3,10]. The degradation temperature (225 °C) of PLA is higher than the melting temperature (170 °C), which is an advantage in polymer processing and composite synthesis, e.g., injecting mold method, in contrast to other bioplastics in which the thermal degradation process (temperature) starts just after the melting temperature and there is a very limited processing temperature window [1]. PLA is also hard and hydrophobic, which makes it suitable for a variety of products [11].

Unfortunately, PLA has some disadvantages that limit its application, like brittle-ness, slow crystallization rate, and low glass transition temperature [12,13].

PLA degradation final products are water and CO_2_, so there is no harmful substance. Compost degradation has two steps. Firstly, the PLA chains are broken, resulting in small polymeric parts and lactic acid. Secondly the microorganisms in the compost mineralize polymeric fragments and generate methene and CO_2_ [14]. The cellulose-based materials can biodegrade without any harmful impact on the environment; in addition, mechanical degradation can be caused, for example, by the action of wind and waves or meso- and microfaunal activities [15].

PLA, like most polymers, is sensitive to UV irradiation, which decreases its lifespan [16]. PLA degrades due to the intervention of weathering factors (water, UV irradiation) [6,17], and the degradation time depends on the degree of crystallinity [11]. That process is impacted by molecular weight and mechanical properties [6]. UV irradiation causes bulk degradation of the main chain of PLA due to C-O bond cleavage via the Norrish II-type mechanism. It manifests in the decrease in mechanical properties and can be detected by spectroscopy due to high absorption at 3290 and 990 cm^−1^ [17,18].

Due to the easy processability of polymers, they commonly play the role of matrix in composite material and easily undergo modification. Their most commonly used modifiers are ceramics [19], carbon materials (carbon nanotubes, graphite, graphene, soot) [20], and other polymers in different shapes and forms [21].

Biofillers have many advantages, starting from physical properties like their low density, recyclability, and CO_2_ naturalness [5]. They are common waste from agriculture, which makes their cost very low in comparison with the most popular fillers that have been extensively investigated during the last years, like carbon nanotubes and other nanofibers and nanoparticles [15]. Also, they are mostly non-toxic, which is an additional advantage over fillers from non-renewable resources [13]. Additionally, PLA is a relatively expensive polymer, so the usage of fillers may reduce the cost of production [22]. The addition of silk, wood flour, various cellulose forms, and wood-based fillers in general may increase the mechanical properties of PLA [1,23,24]. It generates a double-win situation for the environment. It lowers the usage of non-degradable polymers by replacing them with degradable PLA and allows for the use of wood industry waste disposal, which may be troublesome [25]. Worth noting is that lignin (present in wood biofillers) absorbs UV irradiation [26,27], which makes it favorable in the UV protection of polymeric materials. In some works, authors have suggested a modification of biofillers to improve bonding with the matrix [13,28], but this approach requires additional treatment that consumes energy.

There have been numerous studies conducted on the mechanical properties of PLA with cellulose fillers, especially wood fillers. Liu et al. [29] presented research on the mechanical behavior of PLA composites fabricated with FDM (fused deposition modeling)—printing with the addition of wood, ceramic, metal, and carbon. They conducted mechanical tests of untreated samples and demonstrated that wood lowered the mechanical properties. Also, the work presented by Travieso-Rodriguez et al. [30] prepared PLA wood samples through fused filament fabrication. In the presented case, the introduction of wood fibers caused a reduction in the adhesion between fibers and PLA, resulting in an increase in the voids among them and a decrease in mechanical properties. The presented results showed that the amount of additive should be adjusted.

Research presented in the scientific literature mainly concerns biodegradation behavior [31,32,33,34,35], UV blocking [36,37], or basic mechanical property examinations. However, there is still a need to fully understand the degradation process and its implications for the mechanical properties of PLA composites exposed to the natural environment, including sun irradiation.

Therefore, this work aimed to obtain PLA-based biocomposites with the addition of cellulose-based additives and evaluate their properties before and after the UV-light-aging process to determine the effects of each additive on the UV-induced degradation process. The researchers also took care to obtain green composites with possibly the lowest use of chemical reagents and energy consumption and no treatment with fillers so that the examined material could be implemented in the industry.

## 2. Materials and Methods

Polylactide with increased UV resistance in the form of granules was kindly delivered by Nature Works (NatureWorks^®^ Ingeo™ 3251D Injection Grade PLA, Minneapolis, MN, USA). The biopolymer 3251D has a glass transition temperature of 55–60 °C and a crystallite melt temperature of 160–170 °C. This grade of PLA (according to the specification card) exhibits a relative viscosity of 2.5 and a melt flow rate (MFR) of 80 g/10 min (210 °C, 2.16 kg) which is suitable for injection molding. The specific gravity of the PLA used in the research is 1.24.

Crystalline microcellulose (Sigma Aldrich, St. Louis, MO, USA, particle size: 51 µm), flax fibers 2–3 mm (Safilin, Nord-Pas-de-Calais, France, linen roving tex 1000, 20 turns per meter) cut in short fibers, and wood flour from the oak (oak particle 100–300 µm) were used as composite fillers.

The wood flour, flax fibers, and microcellulose were dried at 120 °C for 1 h to remove excess water. The final humidity was about 6.5% for wood flour, 0.2% for microcellulose, and 1% for flax fibers. The composites were prepared using an injection molding process.

Before the injection, the material was homogenized by a single-screw extruder L/D 25 with 4-section temperature control (delivered by ZAMAK, Skawina, Poland) at 175 °C. Using a single-screw extruder, one-step homogenization was implemented (the homogenized material was not homogenized again).

A single-screw extruder was chosen as the homogenization method to investigate the aging effect of UV radiation degradation on PLA/cellulose composites containing cellulose filler aggregates. A single-screw extruder is the most popular method of producing and homogenizing plastics for packaging.

The vertical injection molding machine was produced by Zamak Mercator (Skawina, Poland), and the injection molding process was performed vertically at the top of the sample (parallel to the long side of the sample). After preliminary research, a modifier weight content of 2% was selected by weight. In the mixing process, longer flax fibers in higher concentration could wrap the screw in the plasticizing system and clog the nozzle. Therefore, the amount of 2% was chosen, on the one hand experimentally and according to the literature, on the other hand through our tests using laboratory testing machines.

The granulate obtained in this process may not exhibit the highest homogenization level; nevertheless, in the injection molding process, the produced granulate should lead to the creation of biocomposite with an even distribution of the cellulose-based additive in the bioplastics. In this case, the assumption has been made that the samples would be isotropic. The high-pressure injection molding process causes the one-directional flow of the melted bioplastics and their freezing (crystallization) in the injection mold, so the material should exhibit higher mechanical properties in the injection direction.

The parameters of the process were set to match the type of polymer. The cylinder temperature was 225 °C and the mold temperature was 40 °C. The injection force was 10,000 N and the time was 10 s. A fixed weight of filler was poured into the cylinder and mixed with a melted polymer. The form was covered with a thin layer of releasing agent before each use. Twenty dog-bone-shaped samples were produced for each type of composite and pure PLA.

**Figure 1:**
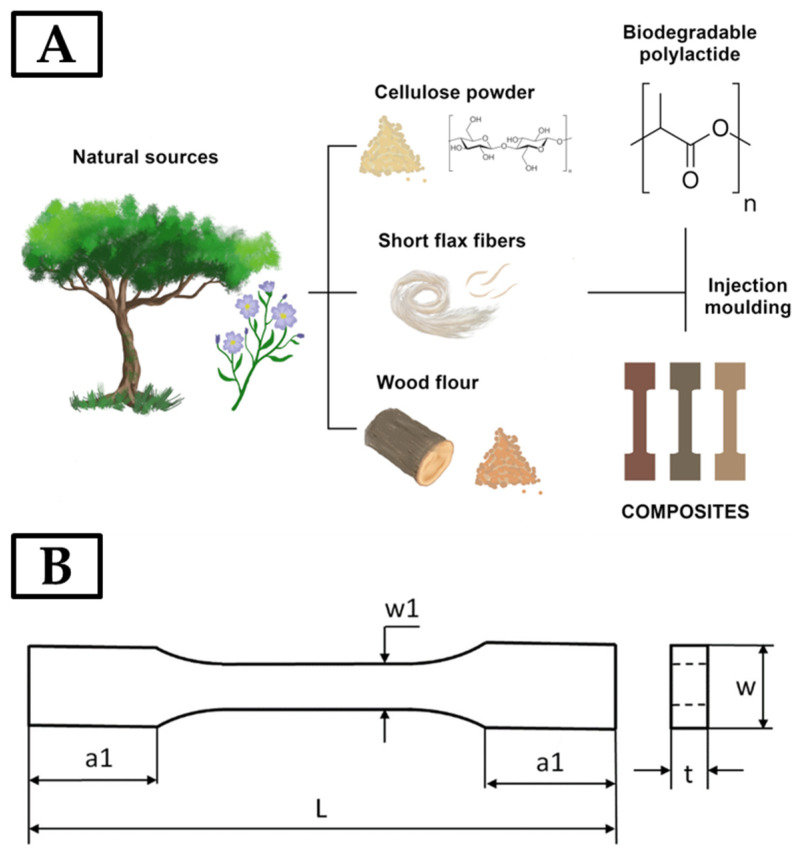
Scheme of obtaining composites with different types of biofillers of plant origin (**A**); sample shape and dimensions (**B**).

The dimensions of the samples were: L = 80 mm, a1 = 15 mm, w1 = 5 mm, w = 10 mm, and mean thickness t = 4 mm (Figure 1B). The mean weights of the samples amounted to 1.6 g (pure PLA) and 1.8–2.1 g (PLA + cellulose). An oil-cooled microtome was used to trim the test samples.

Half of the samples were used in the aging test. Samples were placed in a special chamber constructed by our team with an Hg UV-C 11 V lamp, horizontally at 90° to the UV radiation. Samples were placed on a very thin support made from aluminum; additionally, the chamber walls and bottom were lined with an aluminum mirror in such a way that the UV radiation had access to the samples from all sides and from the bottom [16]. Air circulation was forced to prevent the formation of ozone, which can accelerate the degradation of PLA, from oxygen by UV irradiation. The samples were exposed to UV light for 4 h. The temperature in the chamber, 40 °C, was kept below the glass transition temperature of the PLA. The equivalent exposure time to UV radiation was calculated from the average exposure of the Earth to the Sun’s UV radiation (62 W/m^2^—[19]).

(1) $$\mathrm{Lamp}\;\mathrm{UV}\;\mathrm{exposure}=35\;\frac{W}{{\mathrm{cm}}^{2}}=350\;000\;\frac{W}{m^{2}}$$

(2) $$\mathrm{Earth}\;\mathrm{UV}\;\mathrm{exposure}=62\;\frac{W}{m^{2}}$$

(3) $$\frac{\mathrm{one}\;\mathrm{hour}\cdot \mathrm{Lamp}\;\mathrm{UV}\;\mathrm{exposure}}{\mathrm{Earth}\;\mathrm{UV}\;\mathrm{exposure}}=\frac{1\;h\cdot 350\;000\;\frac{W}{m^{2}}}{62\;\frac{W}{m^{2}}}=5645\;h$$

**Figure 2:**
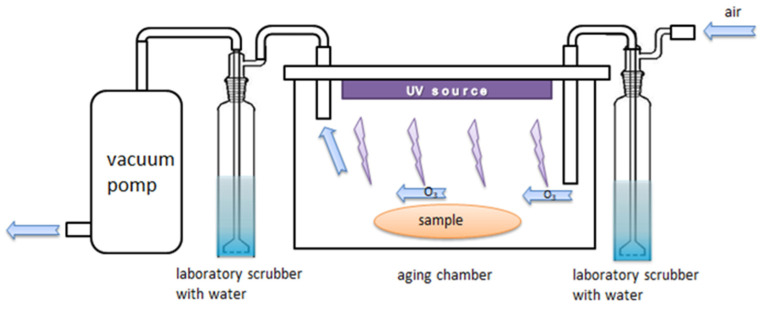
The chamber for UV-aging test, equipped with a UV lamp and a system consisting of a distilled water vessel, pump, and piping system forcing air circulation [19].

According to Equation (3), one hour in the aging chamber equals 5645 h of exposure to solar radiation, with the assumption that the Sun shines continuously and all UV irradiation reaches the Earth’s surface. The samples were exposed to UV light for 4 h (Figure 2).

### 2.1. Mechanical Tests

The external parts of the samples were cut off in a way that was not harmful to the sample, and their shapes remained rectangular. Three-point bending tests were carried out using the Zwick 1435 machine (Skawina, Poland). The conditions and method of performing the bending test of plastics are described in the PN-EN ISO 178 standard [38]. At least 6 samples of each type were tested; the standard requirement is 5. The force acted in the center of symmetrically arranged supports.

(4) $$E=\frac{l^{3}}{{\mathrm{bh}}^{3}}\cdot \frac{\Delta F}{\Delta S}\;\left(\mathrm{Pa}\right),$$

where:

- E—Young’s modulus (Pa),
- l—distance between supports (m),
- b—sample width (Pa),
- h—sample height (m),
- ΔF—change in Force (N),
- ΔS—change in deflection (m), for the initial straight-line portion of the load-deflection curve.

During the three-point bending tests, parameters like the work of destruction, bending strength, and bending strength modulus were established.

For the execution of the impact tests, the Charpy method was used (Charpy hammer Inston 9050). A pendulum of known mass and length was dropped from a known height to impact the sample for the calculation of the work of breaking the material. The work of breaking the material is the difference in potential energies of the pendulum in the initial position and the final position after breaking the sample:

(5) KI=mgR(1−cosα),

(6) KII=mgR(1−cosβ),

(7) K=KI−KII= mgR(cosβ−cosα),

where:

- KI—initial pendulum energy (J),
- KII—final pendulum energy (J),
- m—mass of the pendulum (kg),
- g—standard gravity (m/s^2^),
- R—distance from the axis of the pendulum to the center of the sample (m),
- α—hammer pendulum fall angle,
- β—angle of swing of the pendulum after the sample’s breaking.

### 2.2. Surface Characterization by Optical Microscopy and Roughness Tests

Surfaces of the produced biocomposites and the process of biodegradation on the surface and inside the samples were subjected to microscopic observations using the KEYENCE VHS digital microscope (Keyence, Osaka, Japan).

The characterization of the surface morphology of PLA and PLA with a cellulose-based additive was studied using a scanning electron microscope (Hillsboro, OR, USA, SEM, Nova NanoSEM 200) with an accelerating voltage of 18 kV. Samples were mounted onto special holders and coated with a conductive carbon layer prior to SEM analysis. SEM images were used to compare the surface of the samples and changes that occurred under the UV radiation. SEM observation focused on additions and defect identification. Phase boundaries in the biocomposite were observed.

Roughness tests were carried out on the Hommelwerke T1000 profilometer by JENOPTIK (Jena, Germany). The device measured the roughness on a 4.8 mm section by passing the needle. Three values characterizing roughness were determined: R_a_, R_z_, and R_t_. Ra is the average roughness of a surface, Rz is the difference between the tallest “peak” and the deepest “valley” in the surface, and Rt is a total height of the roughness profile.

### 2.3. Infrared Spectroscopy

The chemical composition of materials before and after the aging test was analyzed using infrared attenuated total reflection spectroscopy (ATR). FTIR measurements were performed using a Bruker Tensor 27 spectrometer (Billerica, MA, USA). Data acquisition was performed in the 4000–600 cm^−1^ spectral range, with 4 cm^−1^ spectral resolution. Sample and background spectra were co-averaged 64 times.

## 3. Results

### 3.1. Mechanical Properties

**Figure 3:**
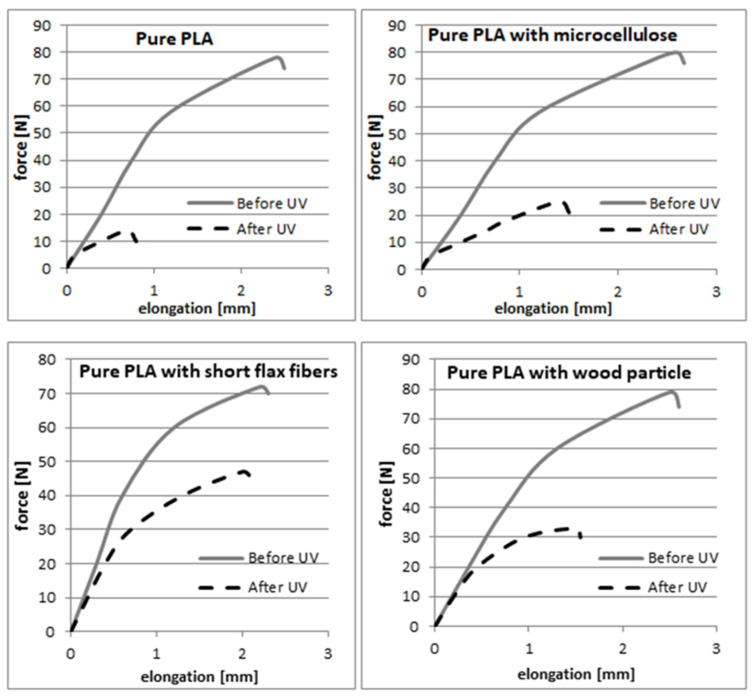
Force–elongation curves of the produced materials before and after the aging process.

The curves in Figure 3 clearly show that during the aging process, the characteristics of the mechanical properties of the investigated composites changed. The samples before aging could hold stress in a similar range—70–80 MPa. In this case, the best performance was observed for microcellulose composites, but the increase in properties was comparatively low (5%). Those experiments concluded that the role and influence of the cellulose additives on the strength of the obtained composites were slight and infeasible. The values obtained from the mechanical tests of the freshly produced samples of both the pure PLA and biocomposites were very similar and fluctuated in the range of +/−8% in relation to the value of the mechanical properties obtained for the pure PLA. We observed an interesting effect during the examination of samples after the aging process: a dramatic decrease in stress and strain. Significantly different impacts on the composite’s properties were revealed depending on the biofiller.

Pure PLA had the lowest value, which proved that the fast degradation and loss of mechanical properties was the most significant. The cellulose particles improved UV-aging resistance, which was observed for the PLA–short flax fiber samples. Additionally, a ~12% decrease in loaded force was observed compared to an unmodified sample made of pure PLA before aging. This was also observed after aging; the PLA–flax fiber samples were the best compared to the other cellulose-based additive samples (60% and 70% decrease for wood flour and microcellulose, respectively). In Table 1, the work of destruction measurement obtained during the bending tests of the samples were collected.

**Figure 4:**
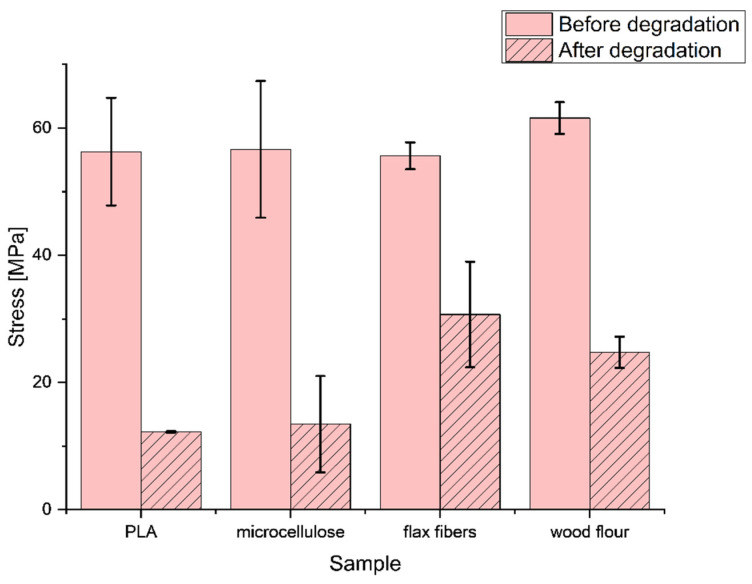
Flexural strength of composites before and after the aging process.

The samples with cellulose-based additives exhibited a slightly lower value of the work of destruction. The work of destruction decreased even more after the aging process in every type of composite sample (Table 1). However, the highest drop was observed for pure PLA. Every cellulose-based modifier slowed down the decrease in the value of the work of destruction during the bending test. The lowest decrease was observed for samples modified with flax fibers, which was 60% in comparison to pure PLA and PLA–microcellulose samples with a decrease of over 80% or PLA–wood flour sample with a decrease of 71%. In Figure 4, the flexural strength results of all the tested samples (before and after the aging process) were presented.

**Figure 5:**
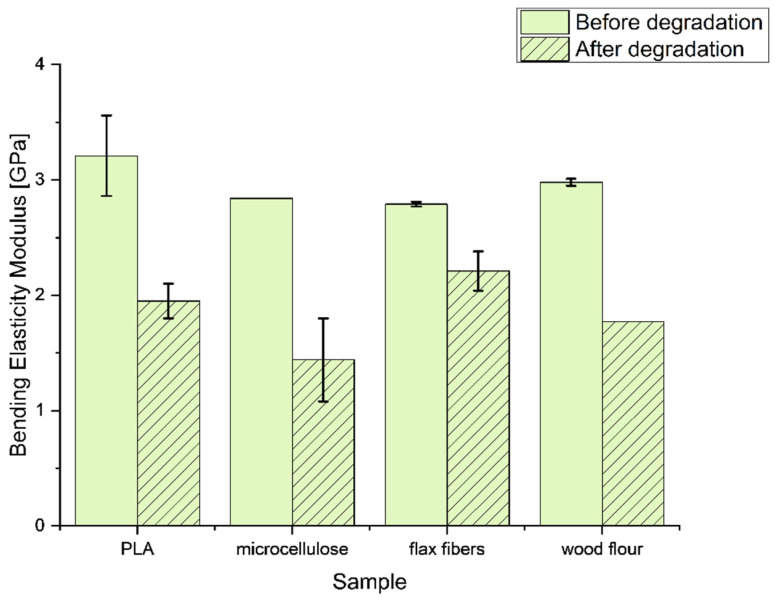
Bending elasticity modulus of composites before and after the aging process.

The tensile strengths of the composite and reference samples were similar to each other, except for the PLA samples with wood particles, where an increase of 9.4% was observed. After UV degradation, the tensile strength of all the samples decreased. The most significant decrease in mechanical strength was observed for the unmodified PLA sample; after UV degradation, the strength value was only 21.7% of the basic tensile strength. Samples containing microcellulose experienced a large decrease in strength values (23.8% tensile strength after degradation). The wood powder protected PLA from degradation; the measured strength was 40.2% of the strength against UV degradation. The highest protection against UV radiation was observed for PLA composites with flax fibers; it amounted to 55.2% of the initial value and, in general, among all the assessed samples, the PLA composite sample modified with flax fibers had the highest tensile strength after UV degradation. In Figure 5, for the bending elasticity, the modulus results from the measurements before and after the aging process are presented. The cellulose-based additives had a slight effect on the bending elasticity modulus, which can be seen in the slight decrease in values compared to pure PLA (Figure 4).

The aging process in every sample caused a decrease in the bending elasticity modulus. All of them were significant, close to 50% of the basic value, with an exception for the flax fiber sample, which prevented the loss of the bending elasticity modulus during the aging process. It was caused by the specific structure of the flax fibers and the consolidation with a bridging effect, which protected the material from stress during the tests.

The lowest values of the elastic modulus for microcellulose resulted from the shape of these particles. Microcellulose is the crystals of small cellulose fibers, which in the produced composite concentrated the stress coming from the mandrel in the testing machine, acting as a defect (hence, pure PLA has a higher value). Elongated modifiers are best for transferring stress because they disperse the accumulated energy into a larger matrix volume.

### 3.2. Impact Mechanical Characterization

**Figure 6:**
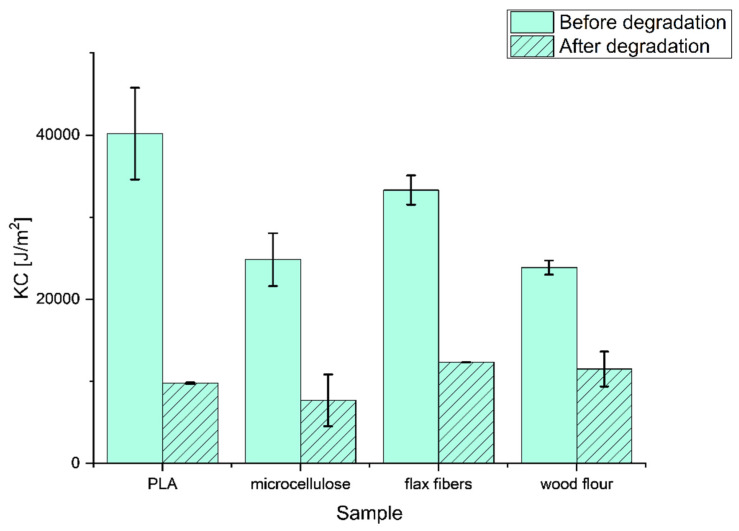
The strength measurement impact before and after the aging process.

In Figure 6, the results of the impact strength measurements of all biocomposite samples before and after the aging process are collected.

The results prove that the actions of UV light and ozone have a significant impact on the decrease in resistance to the dynamic stress of samples. Pure PLA just after preparation had the highest resistivity to the impact in comparison with cellulose-based composite samples. The additives caused a 15% (flax fibers) and 40% (wood flour) decrease in KC. The decrease in the resistance to the dynamic stress could also have been caused by the air that was trapped in the samples in the injection molding process, because even with 10 kN of force on the piston extruder, it was impossible to obtain the pure sample without air disruption.

### 3.3. Investigation of Composites Topography before and after Aging

**Figure 7:**
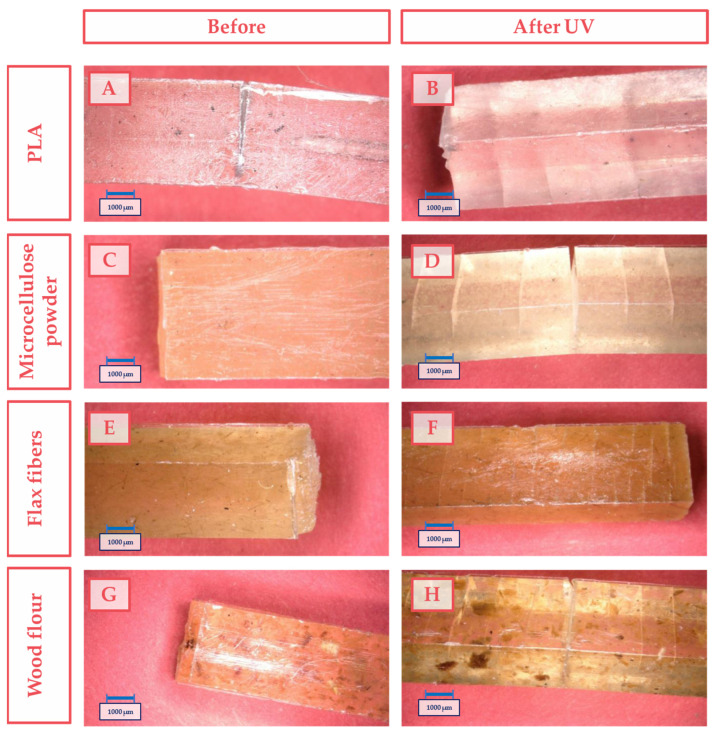
Presentation of an exemplary profile of the surface of the tested composites before aging: (**A**) pure PLA, (**C**) PLA with microcellulose, (**E**) PLA with flax fibers, and (**G**) PLA with wood particles; and after aging: (**B**) pure PLA, (**D**) PLA with microcellulose, (**F**) PLA with flax fibers, and (**H**) PLA with wood particles.

Figure 7 shows an example of a surface profile of pure PLA and the biocomposite before and after UV exposure.

In Figure 7, cracks vertical to the sample length that appeared during the stress can be observed. The cracks occurred at almost the same repeated intervals and were caused because the stress generated during the mechanical test could not be easily dispersed along the length between the support. Such a phenomenon drastically reduced the work of destruction and mechanical parameters of the biocomposites. Particles present in biocomposites were not necessarily aggregates of cellulose additives, but large flakes of cellulose, e.g., from wood. The wood particles in the biocomposites (Figure 7G,H) also contained additives in the form of lignin and hemicellulose, which undoubtedly had an impact on reducing the influence of cellulose on the mechanical properties of the prepared PLA/wood biocomposites. However, lignin, because of its molecular structure, could effectively inhibit/delay the radial UV degradation of the entire composite. The cellulose additives were intentionally not further processed (milled, screened) to reduce the consumption of energy during the manufacturing and to check the effects of commercially available cellulose additives on the PLA matrix.

The samples made of unmodified PLA had an exceptionally smooth surface, the smoothest of all the examined samples. This is because it contained no additives, and the melted material could better fill the form (because of the lowest viscosity). The aging process caused the destruction of the surface microstructure. For composite samples, the quality of the surface before aging was slightly rougher. After the aging process, the degradation of the surface was as high as that in the pure PLA sample. In Table 2, the parameters R_a_, R_z_, and R_t_ of the investigated biocomposites were collected.

**Figure 8:**
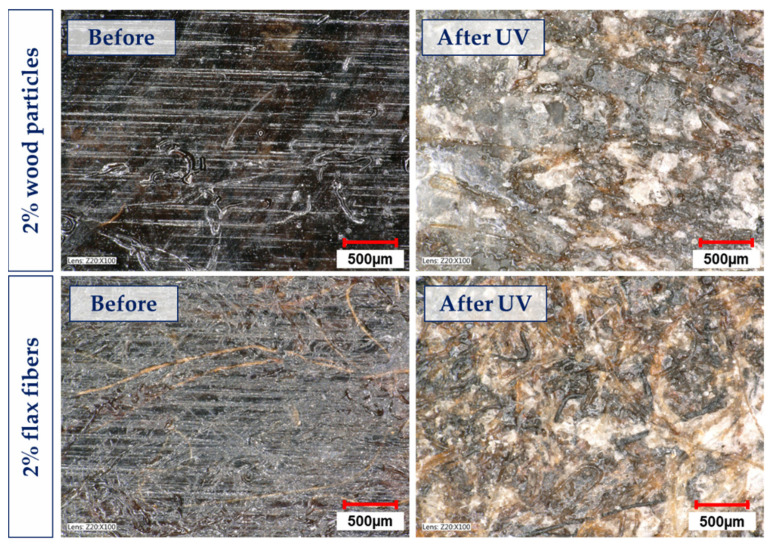
Microscopic photos of the samples: **left**—after the bending test, before the aging process (fresh sample); **right**—after the aging process in the chamber (UV-aged samples).

The obtained results clearly showed that the most degraded surface due to UV influence was the pure PLA sample. Every form of cellulose filler slowed the degradation on the surface and prevented the propagation of critical defects, which cause the disintegration of the material. This was observed on microphotographs (Figure 7). A crucial parameter that may determine the strength of the material is the R_z_ parameter (average of ten highest deviations of a section of the investigated sample). The higher the R_z_, the faster the sample will be destroyed over its whole surface. The decrease in the main roughness parameters after UV aging was the highest for pure PLA, and the lowest decrease was observed for flax fibers and wood flour biocomposites. The microphotographs of these samples before and after aging are shown in Figure 8.

The cracks on the sample surface were present as a result of the bending stress test. The destruction of the material was boosted when the PLA polymer chains shortened; additionally, the presence of defects on the surface in the form of deep scratches was visible. As a result, the material could not hold enough force and could not be used.

**Figure 9:**
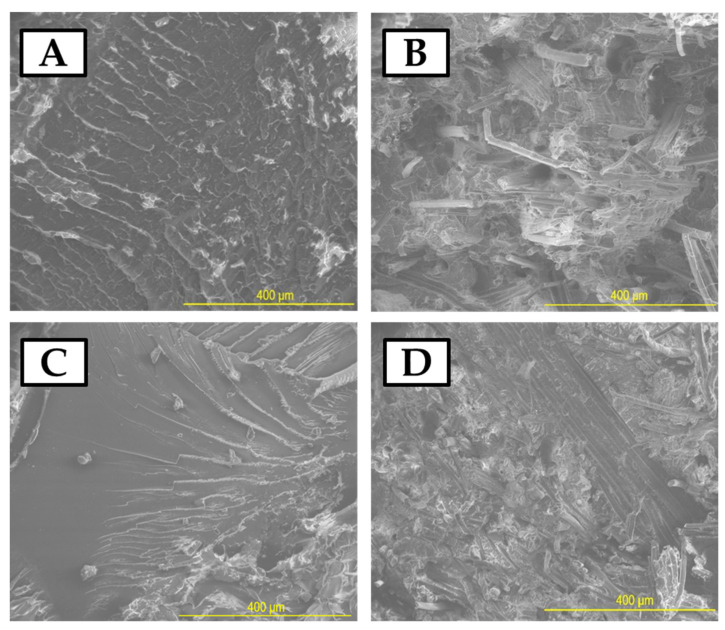
SEM images of samples after Charpy’s test: (**A**) pure PLA, (**B**) PLA with flax fibers, (**C**) pure PLA after UV, and (**D**) PLA with flax fibers after UV.

Figure 9 presents the SEM images of the samples before and after the UV-aging process. There was a significant difference on the surface in the cross-sections of the samples before and after UV aging. On the surface of pure PLA, flow waves of the material were visible because of the high-pressure injection process (similar to microscopic photos in Figure 8). After UV aging, the waves were no longer visible, but microcracks appeared. This was a result of the relaxation process that occurred due to the action of high-energy UV radiation.

After the UV-aging process, the biocomposite samples with flax fibers exhibited the presence of polymer microcrystallites on the surface, which were not present in the material immediately after the manufacturing process (without UV aging).

**Figure 10:**
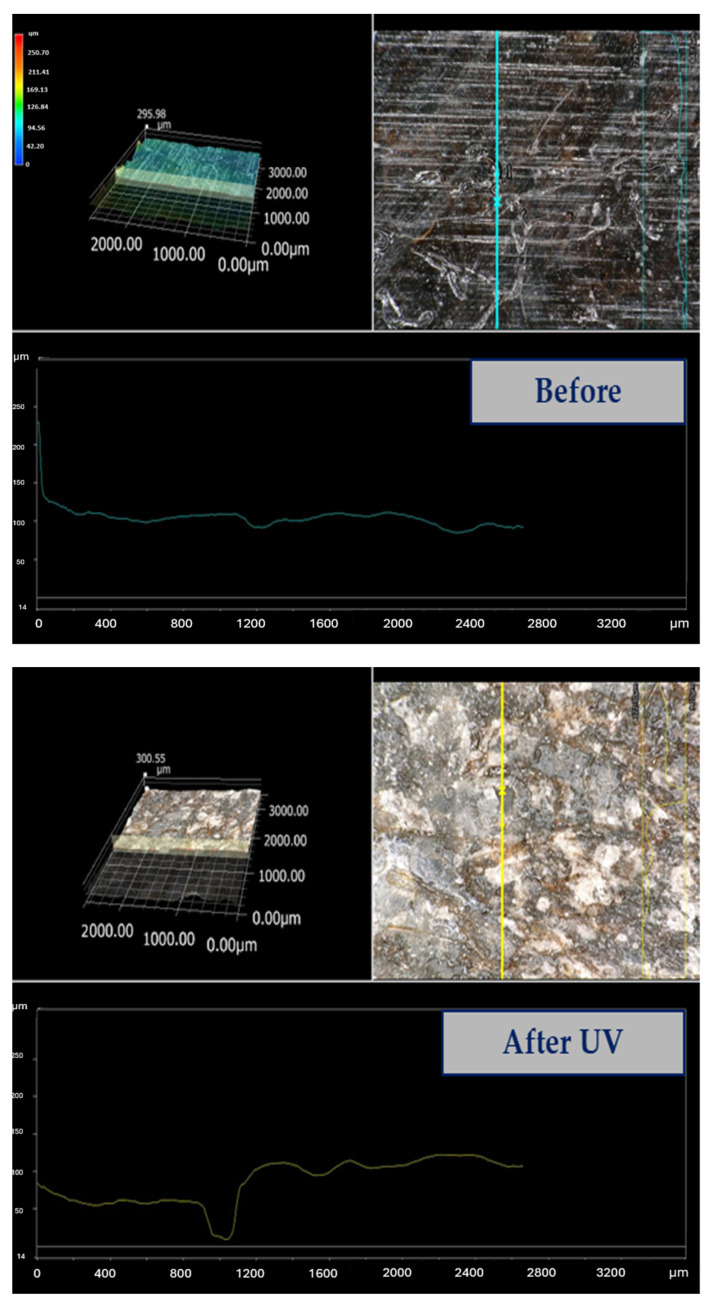
3D profiles before and after aging of the stereoscopic microscopy of the PLA–wood particle sample. The lines in the photos show the place where the sample roughness is measured, the graphs show the measurement of the sample surface roughness.

The surface of the samples was analyzed in 3D with a stereoscopic microscope. Figure 10 shows a comparison of pure PLA before and after aging.

The analysis of the 3D images showed very significant topography changes in the samples. This unequivocally proves that the surface degraded first and at the fastest pace, and that the defects created were responsible for the decrease in the mechanical strength parameters. The main evidence is the surface profile. Before the aging tests, there were small fluctuations, but in the case of the sample after the UV-aging process, there were significant profile changes with a deep hollow.

### 3.4. Structural Characterization

**Figure 11:**
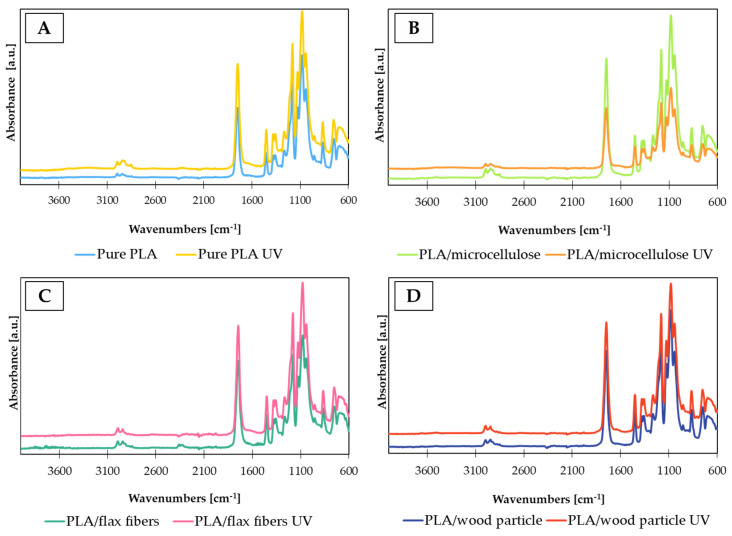
FTIR-ATR spectra of PLA (**A**) and biocomposites with (**B**) microcellulose, (**C**) flax fibers, and (**D**) wood particles before and after UV aging.

The effect of the UV aging of PLA/cellulose UV biocomposites on the chemical structure of PLA-based biocomposites was investigated using the FTIR-ATR method. Figure 11A shows the spectrum of pure PLA. Typical bands of PLA can be found at 2994 cm^−1^ and 2944 cm^−1^ (asymmetric and symmetric stretching vibrations of C-H), 1382 cm^−1^ (symmetric bending vibration of C-H), and 866 cm^−1^ (stretching vibration of C-C) [16]. Another characteristic peak at 1748 cm^−1^ corresponds to the C=O group. Bands at 1181 cm^−1^ and 1080 cm^−1^ can be assigned to the C-O-C vibrations of ester groups [17].

The spectra of the different biocomposites appeared similar to those of pure PLA (Figure 11B–D). The main filler strands may overlap and be covered by polymer matrix strands. Moreover, the selected method (ATR) for testing the impact of photodegradation on the chemical structure of composites takes into account only a thin layer in direct contact with the crystal. the infrared analysis of PLA after the UV-aging test did not show any significant changes in the spectrum or the formation of new bands [17]. It appears that the main degradation reaction was the cleavage of ester groups and fragmentation of side chains into ketones, carboxylic acid, and other products with a C=O band.

## 4. Discussion

The results showed a lower decrease in the mechanical properties of PLA modified with fillers. This was caused by cellulosic additions modifying the PLA matrix. This behavior of the modified material was caused because the modifier (particles) did not work as a reinforcing phase, which prevents and absorbs stress well. The decohesion of material occurs when the stress has a higher value than the strength of chemical bonds in the polymer structure. In this situation, elongated shape additives (like fibers) would be more efficient to reinforce the material and prevent the decohesion. This elongated shape, along with fiber, favors the prevention (the propagation of the crack under the stress during the mechanical tests procedure) of stress at the highest effective volume of the material. Composites containing fibers should exhibit the highest impact strength. Stress observed during dynamic tension is concentrated at the discontinuity of the material (like pores), the presence of which may cause a significant decrease in the strength of the material (it probably happened in this case). The presence of even a small number of pores leads to the concentration of tension, causing the beginning of the material destruction process. Moreover, a decrease in impact strength is caused by factors like the uneven distribution of particles in the matrix and weak adhesion between the ingredients of the composite. Changes are observed after the aging process, which causes degradation of samples on the surface, forming critical defects and leading to the start of destruction. In this case, the reinforcing mechanism of cellulosic additions and their shapes caused a decrease in the impact strength.

The decrease in the mechanical properties of the PLA–cellulose additive samples that were not exposed to UV radiation may have occurred due to the manufacturing process drawbacks. The main reason for the lower values of mechanical parameters may be attributed to the additive’s aggregates.

The aging process is mainly related to the PLA matrix. Bioplastics undergo the hydrolysis process under UV radiation [16]. The addition of flax fibers slows down the process because of the construction and shape of the fibers (long and parallel cellulose chains). The results showed that the greatest losses in mechanical properties after UV aging was for the PLA biocomposites modified with the ultra-clean crystalline microcellulose. The main conclusion from this is that cellulose by itself cannot slow down the aging process; what is more, even the ultra-clean (pure) form of cellulose led to the acceleration of the aging process. This led to the conclusion that the key aspect in this case was the construction and form of the applied cellulose. Even the cellulose “contaminated” with side molecules like lignin and hemicellulose still slowed down the aging process. This led to the conclusion that the main parameter determining the aging process was the surface development of the cellulose additive and the complexity together with the linearity of the added cellulose form.

In comparison to the research on composites based on PLA and carbon additives [39], cellulose-based additives exhibited a weaker delay in the UV-aging process than carbon-based additives because of the different aging mechanisms. Carbon-based additives undergo radiation absorption due to their volume.

## 5. Conclusions

Biocomposites are an excellent alternative for conventional construction composites. Assessed composite is a material that, due to its structure and composition, is completely biodegradable, and decomposition products are safe for the environment. In this paper, cellulose-like additives (varying in shape and source, which determine their properties) and their effect on composite properties were assessed.

It was proven that cellulose-like additives delayed the aging process under UV radiation; thus, the mechanical properties after UV exposure were better compared to pure PLA. It also slowed down the appearance of cracks inside PLA and protected the surface from the appearance of defects, where degradation of the material starts.

The slowest decline in mechanical properties over time was recorded for biocomposites containing flax fibers (decrease of 60%), followed by biocomposites containing wood powder (decrease of 71%). Compared to pure crystalline cellulose (decrease of 81%), these results were significantly lower. The reason for this is the shape of the flax fiber (elongated shape, advantageous for transferring stress) and, in the case of wood powder, the content of lignin (and hemicellulose), which increased the resistance to UV radiation (flax fibers also contain trace amounts of lignin residues, not removed after the combing process and laundry).

The naturally occurring compounds present in cellulose improve the UV resistance of the composite and slow down the aging process of the PLA/cellulose biocomposite. This suggests that the lifetime of these biocomposites under environmental conditions can be elongated because of the slowed process of PLA degradation with the addition of proper cellulose-based additives.

The obtained results are interesting because of the selected homogenization method of the PLA matrix with the cellulose-based modifier in the SSE and injection molding process. This method does not provide the highest level of homogenization like a TSE; nevertheless, it is sufficient for short-term applications (which was proven by the similar mechanical test results).

To obtain better results, the first step is to prepare a material with a higher cellulose-based additive dispersion in the PLA matrix. Better homogenization could also be achieved by using a more advanced plasticizing and mixing system, e.g., using a two-screw extruder (TSE). Another way would be further grinding and homogenizing the cellulose-based additives, but of course these two methods involve higher production costs.

The UV degradation process had a more significant effect on the mechanical properties of the presented materials than the homogenization process. Even for the imperfectly homogenized biocomposites, different types of cellulose additives exhibited an impact on the long-term UV resistance.

The most prominent cellulose additive in terms of delaying the UV degradation process, according to the presented results, was flax fibers. Nevertheless, more tests including a thermal analysis and dynamic mechanical analysis are considered necessary. For food packaging or other constraint applications, a specific test related to the field in which it is found is required.

## Figures and Tables

**Table 1:** Work of destruction for testes samples before and after UV aging.

Material	Work of Destruction (N·mm)	
	Before UV	After UV
PLA	136.56	8.91
Deviation	2.15	0.65
Different	127.65 (88% ↓)	
PLA with flax fibers	120.42	72.78
Deviation	2.04	1.58
Different	47.64 (60% ↓)	
PLA with microcellulose	129.42	24.83
Deviation	2.56	1.43
Different	104.59 (81% ↓)	
PLA with wood flour	131.43	38.53
Deviation	2.14	1.73
Different	92.90 (71% ↓)	

↓ a down arrow indicates a decrease in the value of the measured property compared to the initial values.

**Table 2:** Parameters of Ra, Rz, and Rt of tested biocomposites.

Material	R_a_ μm		R_t_ μm		R_z_ μm	
	Before UV	After UV	Before UV	After UV	Before UV	After UV
PLA	0.25	0.78	2.36	6.06	1.85	4.90
Deviation	0.04	0.09	0.15	0.26	0.11	0.24
Difference	0.53		3.70		3.05	
PLA with flax	0.29	0.53	3.15	4.33	2.65	4.02
Deviation	0.03	0.07	0.17	0.15	0.15	0.23
Difference	0.28		1.18		1.37	
PLA with microcellulose	0.31	0.39	2.01	4.16	1.63	3.34
Deviation	0.04	0.08	0.11	0.13	0.12	0.20
Difference	0.05		2.15		1.71	
PLA with wood flour	0.42	0.81	3.05	5.78	1.99	4.23
Deviation	0.03	0.07	0.13	0.19	0.14	0.19
Difference	0.39		2.73		2.24	

## Data Availability

Data are contained within the article.
